# Supplementary material for: Experimental demonstration of peripherally-excited antenna arrays
Source: Nat Commun. 2021 Oct 20;12:6109. doi: 10.1038/s41467-021-26404-7 (PMC8528866; doi:10.1038/s41467-021-26404-7)
Supplement: Supplementary file 1 — Supplementary Information [file 41467_2021_26404_MOESM1_ESM.pdf]

Supplementary Notes

"Experimental Demonstration of Peripherally-Excited Antenna Arrays"

Ayman H. Dorrah[1] and George V. Eleftheriades[1*]

[1]*The Edward S. Rogers, Sr. Department of Electrical and Computer Engineering,*
University of Toronto, Toronto, Ontario, M5S 3G4, Canada
** e-mail: gelefth@waves.utoronto.ca*

Supplementary Note 1: Phase-Shifting Feeding Network

As discussed in the paper, the proposed 7×7 Peripherally-Excited (PEX) antenna array prototype exhibits 15 peripheral ports along each of the four sides of the structure, and four dummy corner ports. Hence, a feeding network is required to excite one or more sides of the PEX array with appropriately phase-shifted excitations. A 1×16 phase-shifting feeding network is designed, which can be used to excite the proposed PEX array appropriately. A schematic of the designed 1×16 feeding network is shown in Supplementary Figure 1a, and the corresponding fabricated board is shown in Supplementary Figure 1b. It is etched on a double-sided Rogers RT/duroid 5880 1oz substrate with thickness = 0.254mm, and standard SMA connectors are used for the individual RF ports. An additional FR4 1oz substrate is attached to the back of the Rogers board with bond-ply, and it includes the DC bias lines for the individual phase shifters. The phase shifters are implemented using cascaded reflecting hybrid quadrature couplers [1], which are terminated with reversed-biased varactor diodes (voltage-controlled variable capacitors) (see Supplementary Figure 1c). Four M/A-Com MAVR-000120-14110P varactors are used per phase shifter, providing an electronically tuned phase shift by controlling the amount of reverse biasing voltage. As mentioned earlier, the bias voltage lines are routed on the additional FR4 substrate on the back of the phase-shifter board, and connected to the individual varactors using metallic vias. It is worth noting that a DC blocking 10pF capacitor and an effectively RF choke 20kΩ resistor are used to decouple the DC and RF circuits. The DC bias voltages are provided using an external Measurement Computing USB-3114 data acquisition microcontroller that can provide up to 16 analog voltage channels, with a 0-10V bias range. To utilize the full reverse bias voltage range of the M/A-Com varactors which is 0-16V, a non-inverting operational amplifier is used to amplify the input bias voltage supplied by the microcontrollers as shown in Supplementary Figure 1d. For this purpose, Texas Instruments' LM2904DR operational amplifier chips are used.

Two samples of the design were fabricated by Candor Industries Inc and assembled by V.U.nics Inc. Then, the individual phase shifters on the two boards were experimentally tested using an Agilent Technologies E8364B vector network analyzer (VNA). The measured results of both sample boards at the center frequency of 13.1GHz are shown in Supplementary Figure 2. In the absence of material losses and reflections, the maximum transmission should be around

($10\log(1/16) = -12$dB), because the feeding network ideally divides the input power equally between the 16 output ports. The actual measured transmission is substantially lower than the maximum expected value, which can be attributed to material losses of the substrate and copper traces, and additional losses and reflections due to the varactors, RF decoupling resistors, and the RF cables used. This evidently leads to a lower measured realized gain compared to the simulation, as discussed in the paper. Nevertheless, the phase shifters are capable of providing around 497° scan range at the design frequency of 13.1GHz. This scan range is sufficient for demonstrating the full operation of the PEX array. For Boards A and B, phase shifters 3 and 1 are not used to excite the PEX array, respectively, as only 15 phase shifters are required for each side of the array.

The frequency response of both sample boards is shown in Supplementary Figure 3, for the 9th phase shifter on each board. The phase shifting behavior is maintained, while maintaining a stable transmission amplitude throughout the frequency of operation. Overall, the feeding network and phase shifters developed in this section can be used in a flexible and modular manner to excite the proposed PEX antenna array, under single and/or multiple-beam generation modes.

Supplementary Note 2: Frequency Response of the Proposed PEX Antenna Array

In the main body of the paper, full-wave simulation and experimental results of the proposed PEX antenna array are discussed only at the center frequency of operation (13.1GHz). The emphasis there is on the electronic scanning capability of the proposed PEX array as well as the generated pencil beam(s), and not on the frequency response. In this section, the frequency response of the proposed PEX antenna is discussed using both full-wave simulation and experimental results.

Power Distribution The PEX array relies on the excitation of plane wave(s) from one or more of its sides, which are radiated as pencil beams towards various directions in space. It is crucial to investigate the different portions of power reflected, coupled and transmitted between the various sides A, B, C and D of the PEX antenna under various operation scenarios. For the case of single-beam generation, it is sufficient to excite a single side of the PEX array, with a plane-wave along different directions (ψ). The portions of power reaching the 4 sides of a 15×15 PEX array, when only side A is excited, are full-wave simulated and plotted for the entire frequency range of operation in Supplementary Figure 4. The portion of power reflected back to side A is quite small for the various (ψ) directions, especially around the center frequency. At higher frequencies, more power is reflected back for some scenarios, which can be reduced by more careful optimization of the peripheral excitations and/or the radiating unit cells. Additionally, the PEX array exhibits negligible coupling between side A and the adjacent sides B and D throughout the frequency range of operation, and for all the (ψ) directions. On the other hand, a significant portion of the power is transmitted between sides A and C, which can be reduced much further by making the size of the PEX array bigger. Hence, more power is radiated by the PEX antenna, which leaves less remaining power to be transmitted over to side C. Overall, these full-wave simulation results suggest that there are no major concerns with mutual coupling or current cancellation for the proposed PEX

antenna array. Notably, because of the symmetric nature of the proposed PEX antenna, similar results are obtained when the other sides of the array are excited.

Efficiency Simulation The different efficiencies of the proposed PEX antenna are analyzed such as radiation and aperture efficiencies. The radiation efficiency is a metric that characterizes the effect of material (dielectric and conductive) losses of the antenna on the performance, i.e. the gain of the generated pencil beams. The full-wave simulated radiation efficiency of the proposed 15$\times$15 PEX array is depicted vs frequency of operation in Supplementary Figure 5a, for various plane-wave directions (ψ). For all the cases, a radiation efficiency higher than 70$\%$ is achieved, which is quite typical for similar travelling-wave antennas. On the other hand, the aperture efficiency is a metric which compares the directivity of the generated pencil beams from an antenna, to that of a corresponding beam generated by a uniformly illuminated aperture with the same physical size. A higher aperture efficiency means that the antenna is capable of generating narrow beams, from a compact antenna, saving valuable real-estate. The full-wave simulated aperture efficiency of the proposed 15$\times$15 PEX array is depicted vs frequency of operation in Supplementary Figure 5b, for various plane-wave directions (ψ). Relatively high aperture efficiency levels are observed especially for the pencil beams close to broadside, which shows that the PEX antenna aperture is effectively illuminated by the peripheral sources.

Single-Beam Simulation At its heart, the proposed PEX antenna array is a travelling-wave antenna, which exhibits a frequency-dependent 2D dispersion relation. Hence, when the frequency of operation is swept, the generated pencil beams are expected to undergo slight scanning in space. This is highlighted in Supplementary Figures 6-7, which demonstrate the full-wave simulated E-plane and H-plane radiation patterns generated by the 15$\times$15 PEX antenna, for various directions of (ψ) with respect to the x-axis. The overall beam-pointing directions of the generated pencil beams are stable and maintained throughout the frequency range of operation. Additionally, the full-wave simulated peak realized gain achieved by these pencil beams is summarized in Supplementary Figure 8 against the frequency of operation, showing that the generated beams maintain a stable operation with frequency, with no beam splitting or cancellation problems.

Single-Beam Measurement On the other hand, the measured Az and El radiation patterns of the proposed 7$\times$7 PEX array are plotted in Supplementary Figures 9-10 when only side A is excited, and in Supplementary Figures 11-12 when only side B is excited, for various directions (ψ). Notably, the losses and reflections due to the feeding network and RF cables are calibrated out from the measured radiation patterns, by characterizing their performance separately, and removing their effect from the measured patterns using simple post processing techniques. It seems that the measured results in Supplementary Figures 9-12 experience slightly more spatial scanning with frequency when compared with the full-wave simulation results in Supplementary Figure 6-7. Nevertheless, this discrepancy looks more significant than it actually is because the simulated gain patterns are plotted for an angular span of $\pm 180^o$, but the measured results are only plotted for an angular span of $\pm 70^o$, which amplifies the spatial scanning of the measured results. In any case, the measured PEX array experiences additional spatial scanning as it is excited with phase shifters

that exhibit a slight dispersive amplitude/phase transmission response with frequency. Whereas, the simulated PEX array is fed with a constant progressively phase-shifted excitation without any amplitude/phase dispersive effects. However, it is observed that the overall beam-pointing directions of the measured pencil beams are stable and maintained throughout the frequency range of operation, similar to the full-wave simulation results. On the other hand, the measured peak realized gain and directivity achieved by these pencil beams is summarized in Supplementary Figure 13 against the frequency of operation. Similar to the simulation, the generated beams maintain a stable operation with frequency, with no beam splitting or cancellation problems.

Supplementary Note 3: Additional Multiple-Beam Measurements

The concept of superposition can be applied to the proposed PEX array, where multiple sides of the array are excited simultaneously, with plane waves at various directions (ψ). As a result, the PEX array generates multiple pencil beams that are independently scanned as demonstrated in Supplementary Figures 14-15. These measured case of multiple-beam generation are achieved by simultaneous excitation of sides A and B, with appropriate plane waves. Indeed, two pencil beams are generated in all these cases, each exhibiting beam-pointing directions that are independently controlled by the peripheral sources. This helps demonstrates the versatility of the proposed PEX antenna, where the same radiating aperture is shared, and can be excited using different sides, which essentially constitutes multiple antennas in a single shared radiating aperture, saving valuable real estate. Hence, the PEX antenna can be potentially deployed in multiple-beam applications (MIMO) or even duplex systems with simultaneous transmit and receive from the same antenna.

Supplementary Note 4: Spherical Coordinate Systems for Antenna Characterization

Antenna characterization using full-wave simulations or anechoic chamber measurements are carried out to quantify the performance parameters of an antenna, such as, gain, radiation patterns, directivity, cross-pol. These parameters are typically gathered over a sphere, as a function of position relative to the antenna-under-test (AUT). However, the choice of coordinate system used directly affects the mapping of the data to 2D cuts, rendering it beneficial to understand the details of the spherical coordinate system employed.

Every spherical coordinate system exhibits a natural pole in some predefined direction. This stems from "Gauss's Theorema Egregium" which states that it is impossible to cover the surface of any sphere with a finite-sized piece of paper [2]. Along this pole, the AUT undergoes no change in its pointing angle in space, when one of the two defined axes is rotated, i.e. the pole is a singularity. The two spherical coordinates systems of interest to this paper are the Theta-Phi (θ-ϕ) and the Az-over-El (Az/El) coordinate systems as shown in Supplementary Figure 16[3]. Note that antenna parameters plotted using these coordinate systems exhibit identical behaviors along the cardinal planes (x-z and y-z planes) (see bold planes in Supplementary Figure 16). Nevertheless,

the parameters can possibly seem slightly different when they are plotted further away from the cardinal planes, and the difference is intensified the farther away the parameters are plotted from the cardinal planes. It is important to note that the antenna parameters remain unchanged regardless of the choice of coordinate system. It is merely the spherical projection of a sphere onto a flat piece of paper that distorts the perceived image, even if the image itself is unchanged.

In Supplementary Figure 16, each coordinate system has two angles and one pole, where the first angle is measured relative to the pole axis. Thus, a complete rotation along the first angle must go through the pole, and the size of its circle is always fixed. On the other hand, the second angle moves around the pole, and the size of the circle formed is a function of the first angle. Table 1 summarizes the angles and poles for both spherical coordinate systems of interest to this paper [3]. It is important to highlight that throughout the paper, all full-wave simulation results are presented on the (θ-ϕ) spherical coordinate system, whereas all experimental results are presented on the (Az/El) spherical coordinate system.

Table 1: **Spherical Coordinate Systems.** Summary of the angles and poles of the spherical coordinate systems used in the paper.

Coordinate System	Pole	1st Angle	2nd Angle
θ-ϕ	z-axis	θ	ϕ
Az/El	y-axis	El	Az

An illustration of a pencil beam generated at an arbitrary angle and plotted using the Theta-Phi (θ-ϕ) coordinate system is included in Supplementary Figure 17a. For this system, (ϕ) is the azimuthal angle measured from the x-axis, whereas (θ) is the elevation angle measured from the z-axis. The corresponding E-plane (blue) contains the direction of the peak of the beam and the electric field, whereas the H-plane (red) contains the directions of the peak of the beam and the magnetic field. An additional angle (γ) is defined along the E-plane with zero at the peak of the pencil beam. On the other hand, the same descriptive pencil beam is plotted in Supplementary Figure 17b using the Az-over-El (Az/El) coordinate system, where Az is measured along planes parallel to the x-z plane with zero towards the z-axis, whereas El angles are measured along the orthogonal planes that go through the y-axis with zero towards the z-axis. Note that both (θ-ϕ) and (Az/El) spherical coordinate systems are identical along the cardinal planes (x-z and y-z planes). In particular, for the x-z plane ($\phi = 0^o$) the elevation angle (θ) is identical to Az, whereas for the y-z plane ($\phi = 90^o$) the elevation angle (θ) is identical to El. Thus, both coordinate systems can be used interchangeably along or around the cardinal planes.

Supplementary References

1. Pozar, D. M. *Microwave engineering* (John Wiley & Sons, 2009).

2. Gauss, C. F. *General investigations of curved surfaces of 1827 and 1825* (Princeton university library, 1902).

3. Masters, G. F. & Gregson, S. F. Coordinate system plotting for antenna measurements. In *AMTA Annual Meeting & Symposium* (2007).

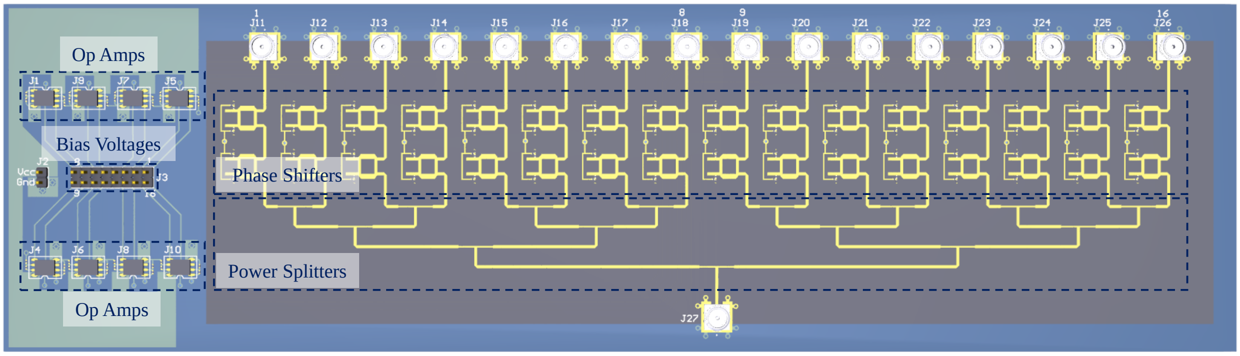

(**a**) Feeding Network Schematic

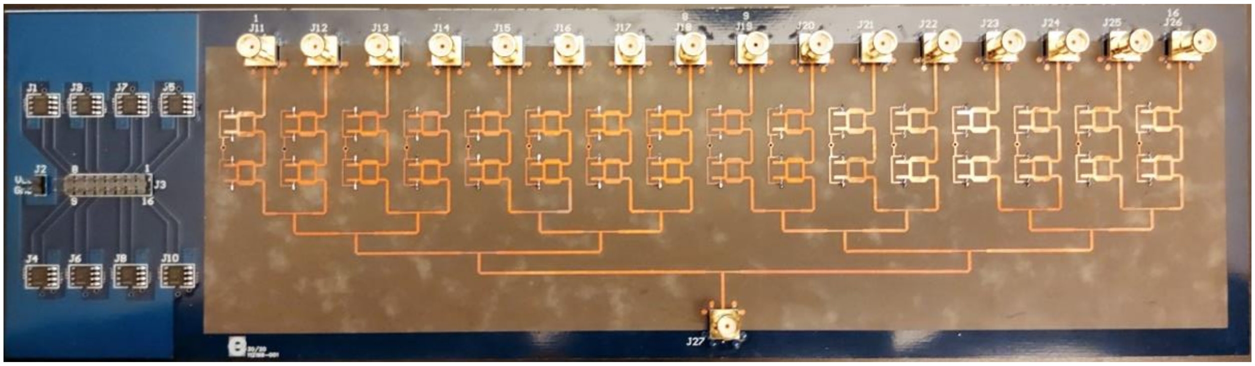

(**b**) Fabricated Feeding Network

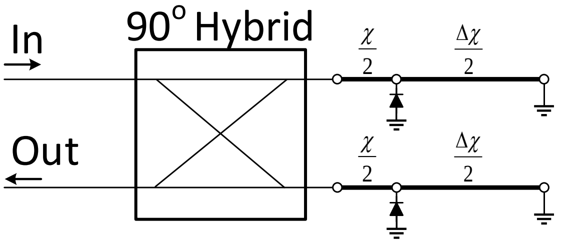

(**c**) Reflecting Quadrature Coupler Phase Shifter

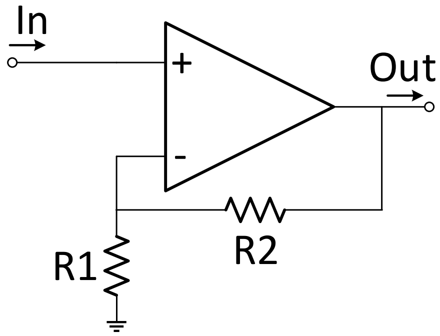

(**d**) Non-inverting Operational Amplifier

Supplementary Figure 1: Phase-Shifting Feeding Network. The developed feeding network with phase-shifters used to excite the proposed PEX antenna array: **(a)** The schematic of the feeding network designed with power splitters, phase shifters, non-inverting amplifiers, and a DC bias voltage header. **(b)** The corresponding fabricated 1×16 feeding network etched on a Rogers RT/duroid 5880 1oz substrate, with DC bias lines on an FR4 1oz substrate on the back. The designed board includes a 2×8 male header that is connected to 8 non-inverting op-amp chips (two op-aps per chip), which amplify the bias voltage and reverse bias the varactors. The power splitters are constructed from a corporate-fed 1×16 power divider that feeds the individual phase shifters (Board Dimensions = 307mm×86.5mm). **(c)** A schematic of the cascaded reflecting quadrature hybrid couplers phase-shifter, which controls the transmission phase of the signal by changing the reverse biased voltage of the varactors. **(d)** A schematic of the non-inverting operational amplifier circuit, which amplifies the bias voltage from the microcontroller, to fully utilize the 0-16V reverse bias range of the varactors ($\text{Out}/\text{In} = 1 + R_2/R_1$).

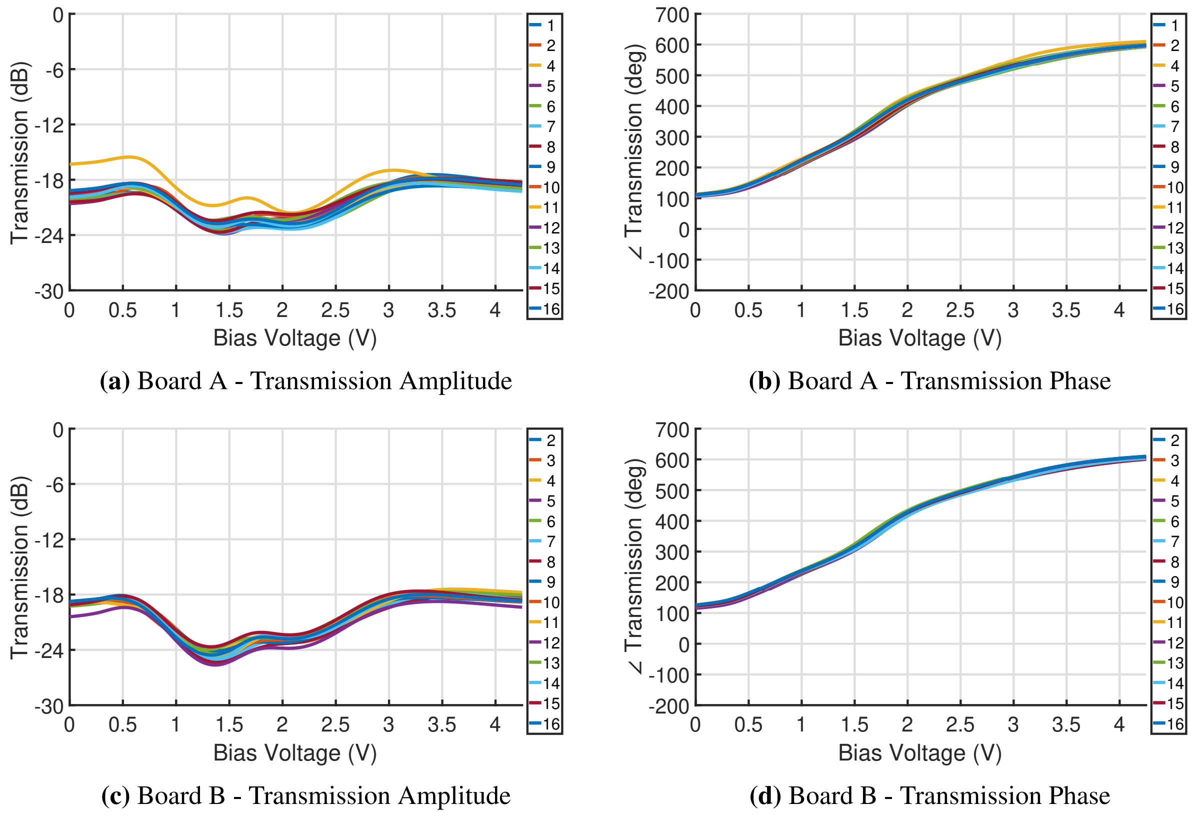

(a) Board A - Transmission Amplitude

(b) Board A - Transmission Phase

(c) Board B - Transmission Amplitude

(d) Board B - Transmission Phase

Supplementary Figure 2: Phase Shifters Scan-Range Measurement. The transmission amplitudes and phases for all the phase shifters on Boards A and B, at the center frequency of 13.1GHz. Around a 497° phase range is achieved by the phase shifters. **(a)**, **(b)** Measured transmission amplitudes and phases of the phase shifters on Board A (phase shifter 3 is unused). **c)**, **(d)** Measured transmission amplitudes and phases of the phase shifters on Board B (phase shifter 1 is unused).

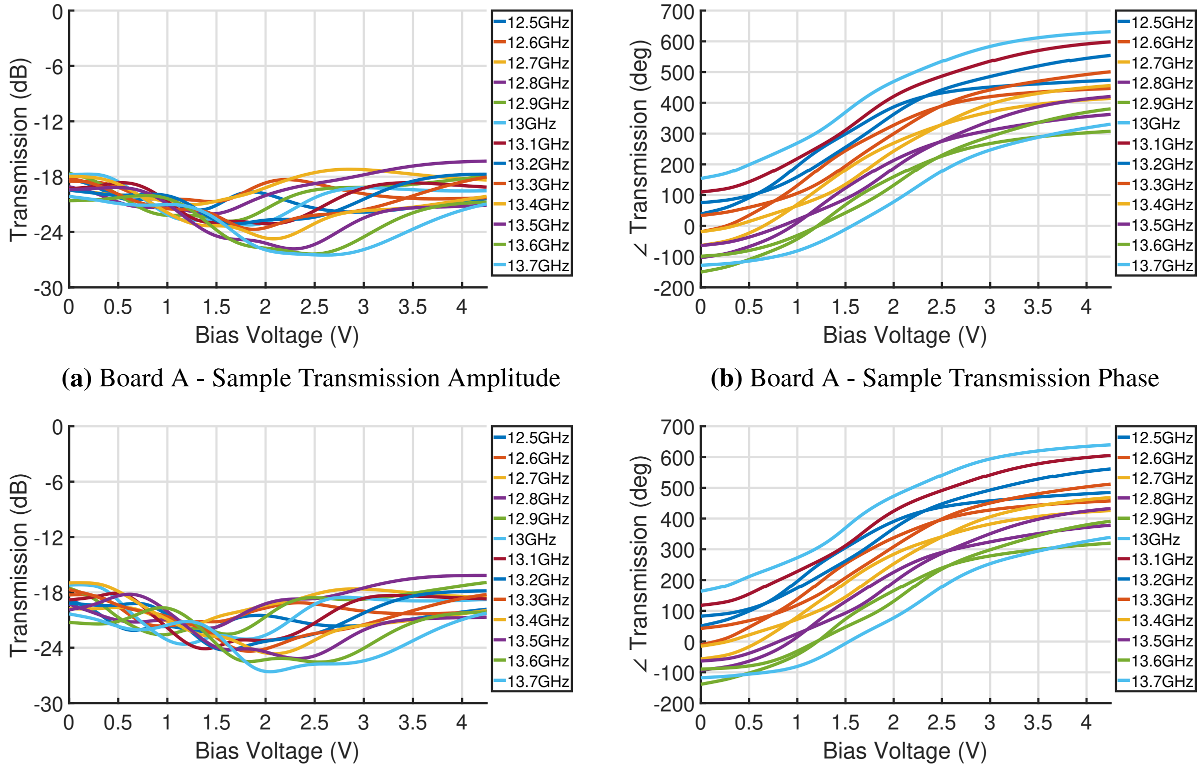

(a) Board A - Sample Transmission Amplitude

(b) Board A - Sample Transmission Phase

(c) Board B - Sample Transmission Amplitude

(d) Board B - Sample Transmission Phase

Supplementary Figure 3: Phase Shifters Frequency-Response Measurement. The measured frequency response of the transmission amplitudes and phases for the 9th phase shifter on Boards A and B. The wide-scan phase range is maintained throughout the entire frequency range of operation. **(a)**, **(b)** Measured frequency response of the transmission amplitudes and phases of the 9th phase shifter on Board A. **(c)**, **(d)** Measured frequency response of the transmission amplitudes and phases of the 9th phase shifter on Board B.

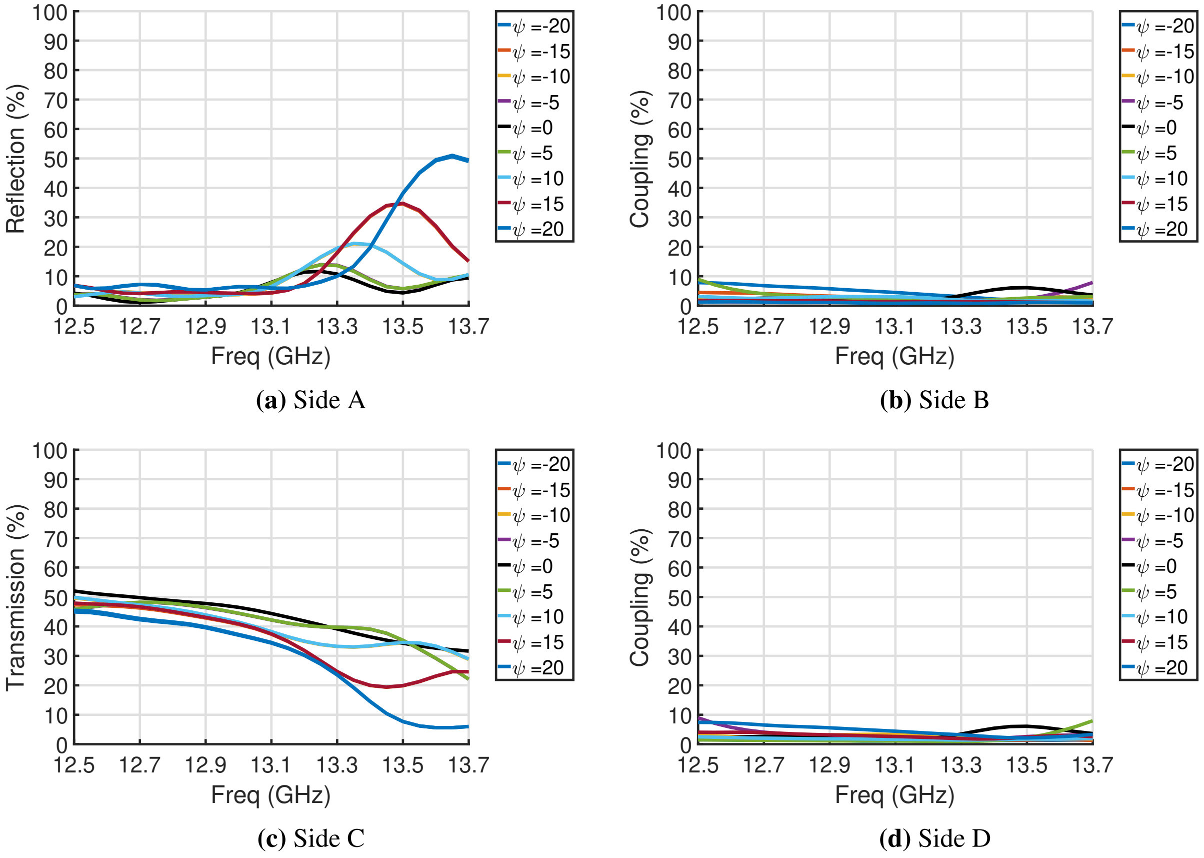

(a) Side A

(b) Side B

(c) Side C

(d) Side D

Supplementary Figure 4: PEX Array Power Distribution. The full-wave simulated power distribution across a 15×15 PEX antenna array vs frequency, for various values of (ψ), when only side A is excited. No problems with current cancellation or mutual coupling are observed. **(a)** The portion of power reflected back to side A vs frequency, for various values of (ψ). **(b)** The portion of power coupled to the adjacent side B vs frequency, for various values of (ψ). **(c)** The portion of power transmitted across the PEX array to side C vs frequency, for various values of (ψ). **(d)** The portion of power coupled to the adjacent side D vs frequency, for various values of (ψ).

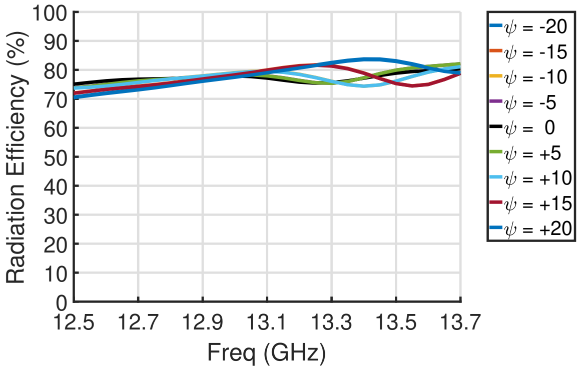

(a) Radiation Efficiency

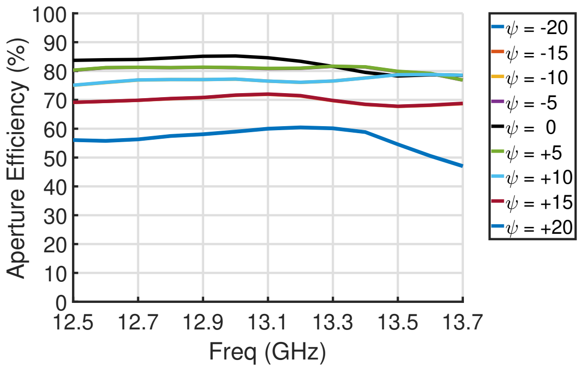

(b) Aperture Efficiency

Supplementary Figure 5: PEX Array Efficiency Simulation. The full-wave simulated radiation and aperture efficiencies vs the frequency of operation of the 15×15 PEX array. The efficiencies are plotted for various values of (ψ), when only side A of the PEX array is excited. **(a)** The full-wave simulated radiation efficiency of the PEX antenna, which characterizes the material (dielectric and conductive) losses of the structure. **(b)** The full-wave simulated aperture efficiency of the PEX antenna, which compares the achieve directivity by the antenna to that achieved by a uniformly illuminated aperture with the same physical size.

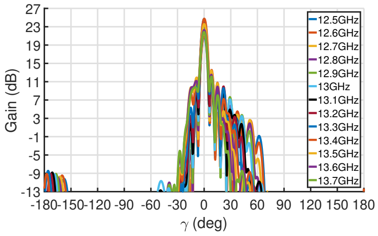

(a) $\psi = -20^o$

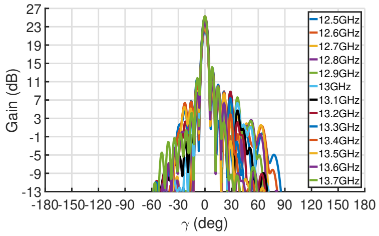

(b) $\psi = -15^o$

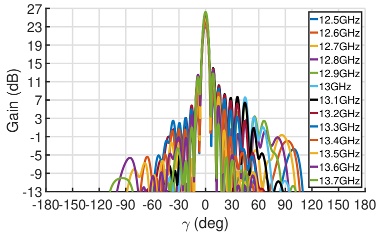

(c) $\psi = -10^o$

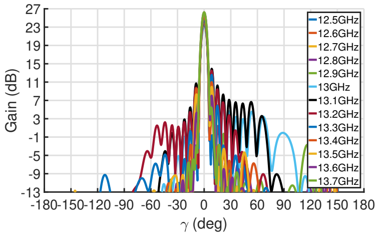

(d) $\psi = -5^o$

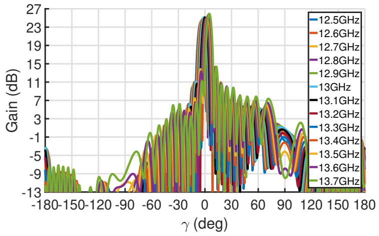

(e) $\psi = 0^o$

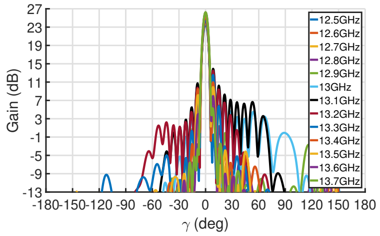

(f) $\psi = +5^o$

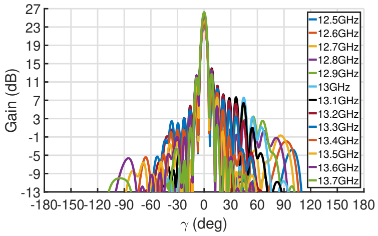

(g) $\psi = +10^o$

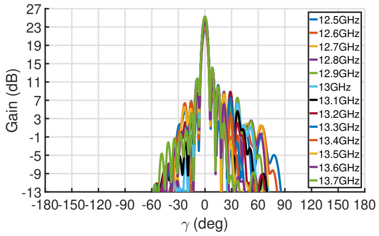

(h) $\psi = +15^o$

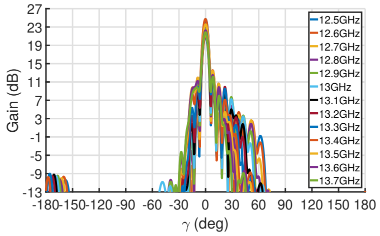

(i) $\psi = +20^o$

Supplementary Figure 6: Single-Beam E-Plane Radiation Patterns Simulation. The full-wave simulated realized gain patterns plotted along the E-plane, for the frequency range of operation of the 15×15 PEX array, for various values of (ψ), when only side A of the PEX array is excited. A stable frequency response is observed for the different excited plane-wave directions (ψ).

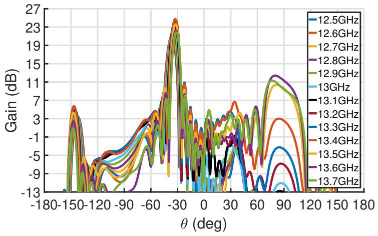

(a) $\psi = -20^o$

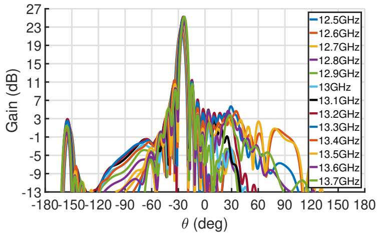

(b) $\psi = -15^o$

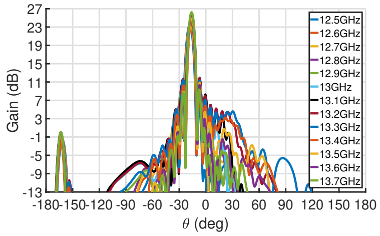

(c) $\psi = -10^o$

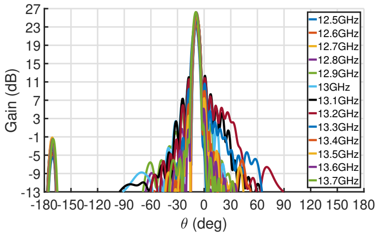

(d) $\psi = -5^o$

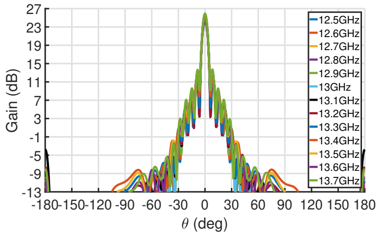

(e) $\psi = 0^o$

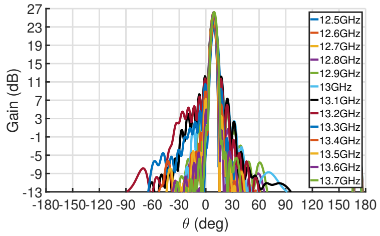

(f) $\psi = +5^o$

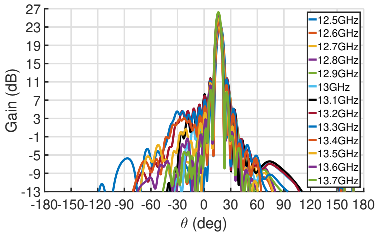

(g) $\psi = +10^o$

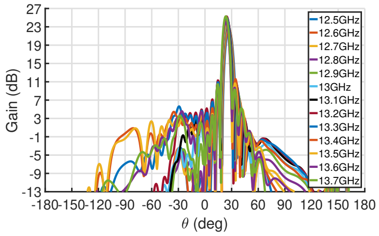

(h) $\psi = +15^o$

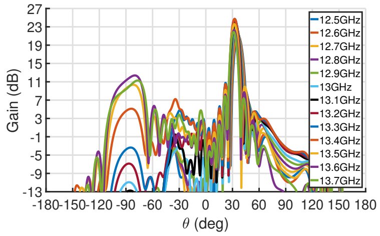

(i) $\psi = +20^o$

Supplementary Figure 7: Single-Beam H-Plane Radiation Patterns Simulation. The full-wave simulated realized gain patterns plotted along the H-plane, for the frequency range of operation of the 15×15 PEX array, for various values of (ψ), when only side A of the PEX array is excited. A stable frequency response is observed for the different excited plane-wave directions (ψ).

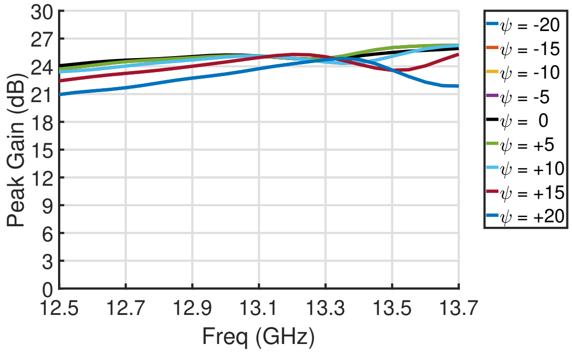

Supplementary Figure 8: Peak Gain Simulation. The full-wave simulated peak realized gain vs the frequency of operation of the 15×15 PEX array. The peak gain is plotted for various values of (ψ), when only side A of the PEX array is excited.

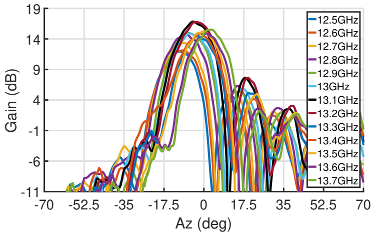

(a) $\psi = -20^o$

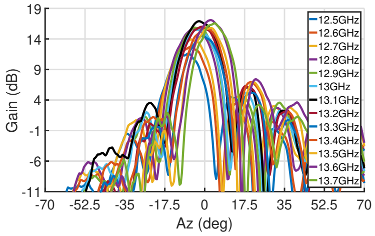

(b) $\psi = -15^o$

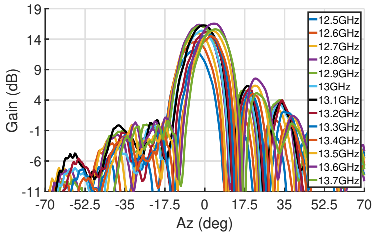

(c) $\psi = -10^o$

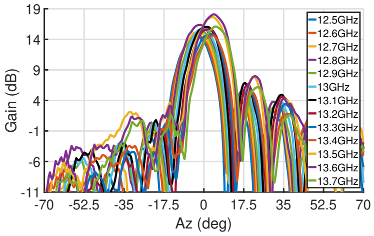

(d) $\psi = -5^o$

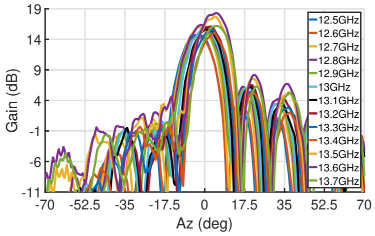

(e) $\psi = 0^o$

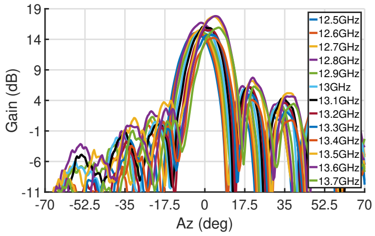

(f) $\psi = +5^o$

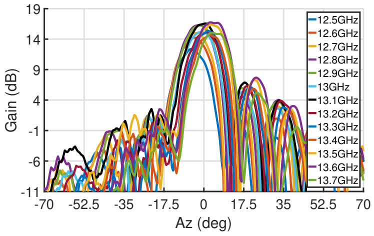

(g) $\psi = +10^o$

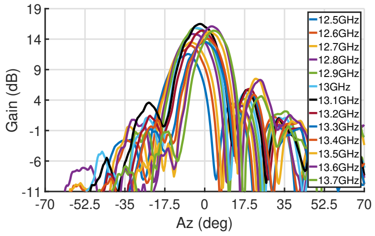

(h) $\psi = +15^o$

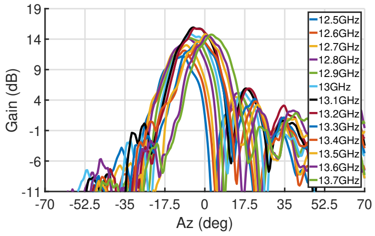

(i) $\psi = +20^o$

Supplementary Figure 9: Single-Beam Az Radiation Patterns Measurement (Side A). The measured realized gain patterns plotted along the Az direction, for the frequency range of operation of the 7×7 PEX array, for various values of (ψ), when only side A of the PEX array is excited. A stable frequency response is observed for the different excited plane-wave directions (ψ).

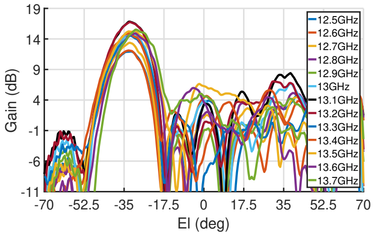

(a) $\psi = -20^o$

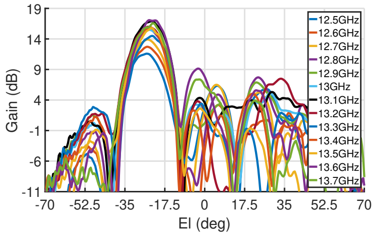

(b) $\psi = -15^o$

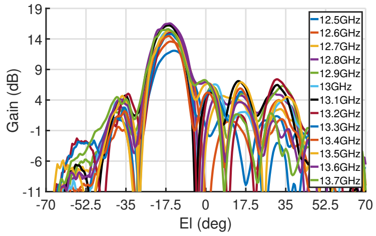

(c) $\psi = -10^o$

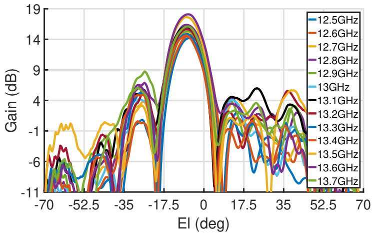

(d) $\psi = -5^o$

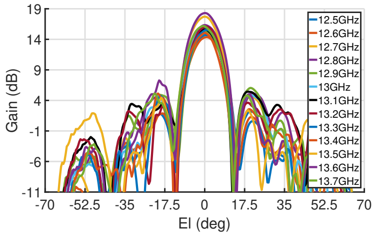

(e) $\psi = 0^o$

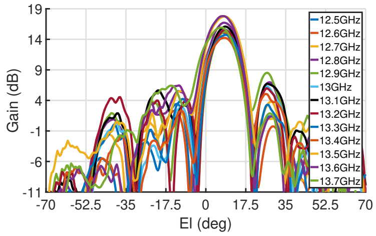

(f) $\psi = +5^o$

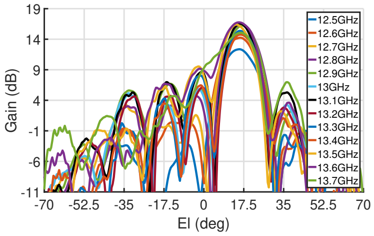

(g) $\psi = +10^o$

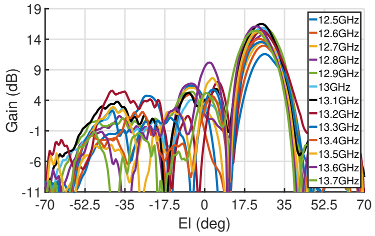

(h) $\psi = +15^o$

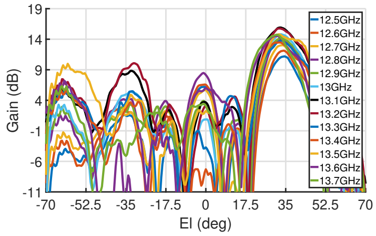

(i) $\psi = +20^o$

Supplementary Figure 10: Single-Beam El Radiation Patterns Measurement (Side A). The measured realized gain patterns plotted along the El direction, for the frequency range of operation of the 7×7 PEX array, for various values of (ψ), when only side A of the PEX array is excited. A stable frequency response is observed for the different excited plane-wave directions (ψ).

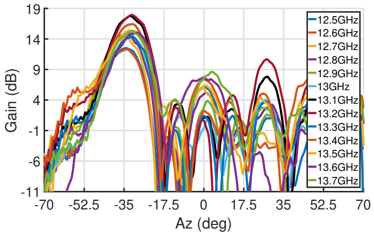

(a) ψ =250°

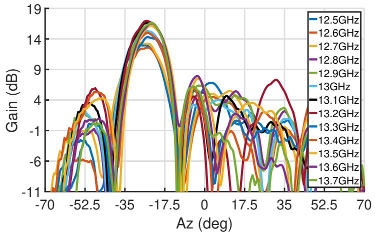

(b) ψ =255°

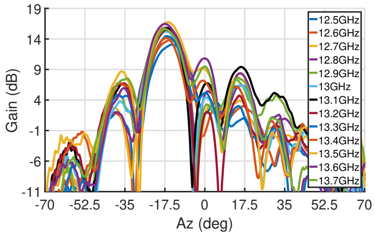

(c) ψ =260°

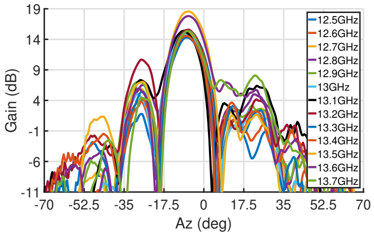

(d) ψ =265°

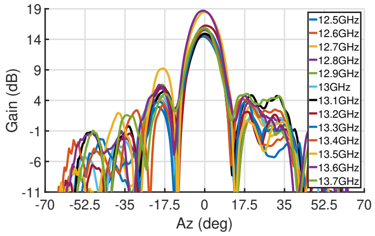

(e) ψ =270°

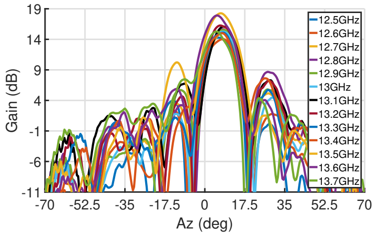

(f) ψ =275°

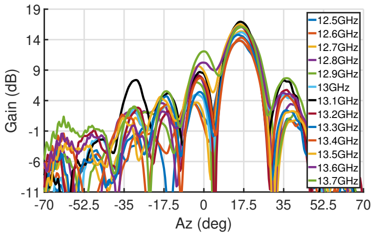

(g) ψ =280°

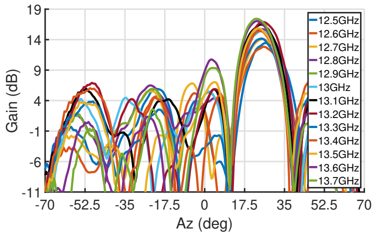

(h) ψ =285°

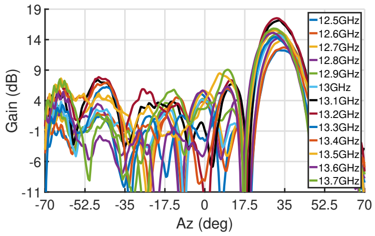

(i) ψ =290°

Supplementary Figure 11: Single-Beam Az Radiation Patterns Measurement (Side B). The measured realized gain patterns plotted along the Az direction, for the frequency range of operation of the 7×7 PEX array, for various values of (ψ), when only side B of the PEX array is excited. A stable frequency response is observed for the different excited plane-wave directions (ψ).

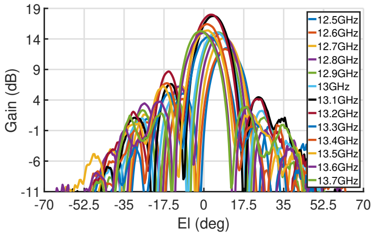

(a) ψ =250°

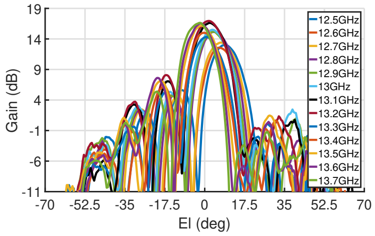

(b) ψ =255°

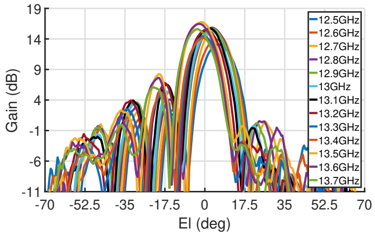

(c) ψ =260°

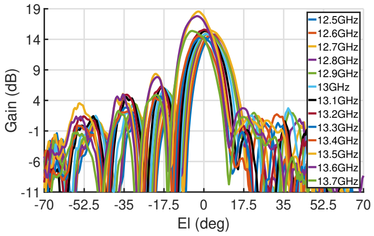

(d) ψ =265°

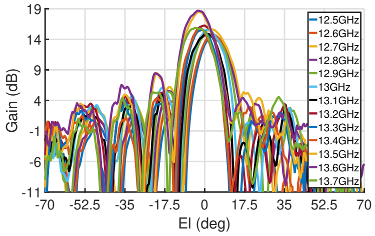

(e) ψ =270°

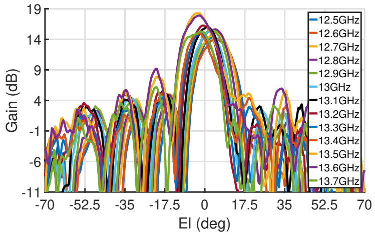

(f) ψ =275°

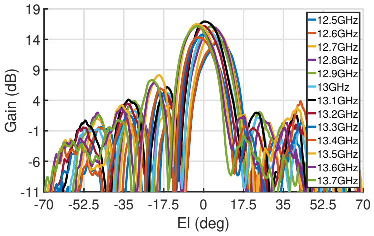

(g) ψ =280°

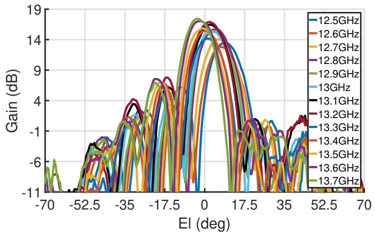

(h) ψ =285°

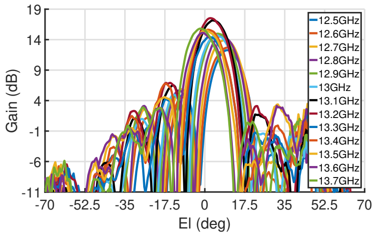

(i) ψ =290°

Supplementary Figure 12: Single-Beam El Radiation Patterns Measurement (Side B). The measured realized gain patterns plotted along the El direction, for the frequency range of operation of the 7×7 PEX array, for various values of (ψ), when only side B of the PEX array is excited. A stable frequency response is observed for the different excited plane-wave directions (ψ).

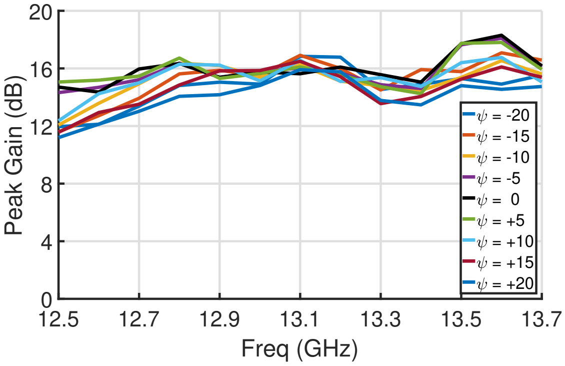

(a) Peak Gain - Side A

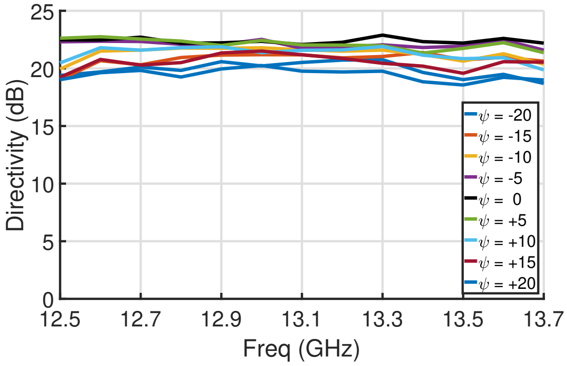

(b) Directivity - Side A

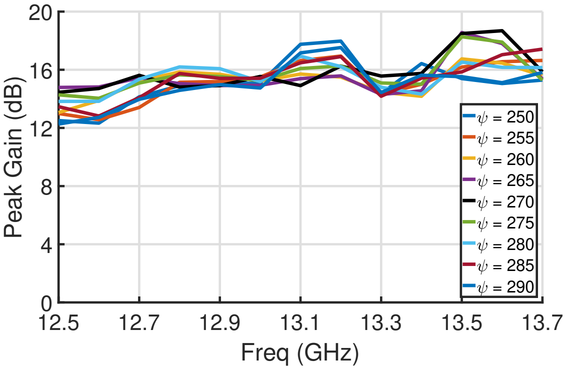

(c) Peak Gain - Side B

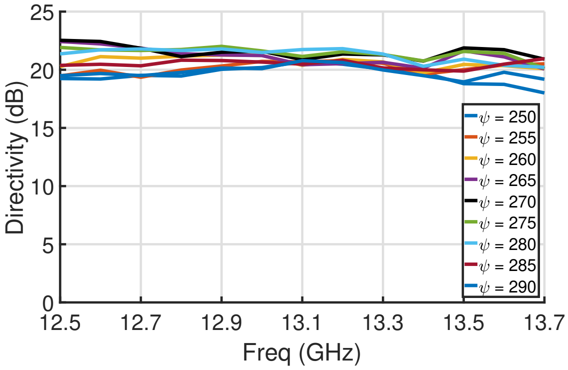

(d) Directivity - Side B

Supplementary Figure 13: Peak Gain and Directivity Measurement. The measured peak realized gain and directivity vs the frequency of operation of the PEX array. They are plotted for various values of (ψ), when either side A or side B of the 7×7 PEX array is excited. **(a)**, **(b)** The measured peak gain and directivity vs frequency of the PEX array, for various values of (ψ), when only side A is excited. **(c)**, **(d)** The measured peak gain and directivity vs frequency of the PEX array, for various values of (ψ), when only side B is excited.

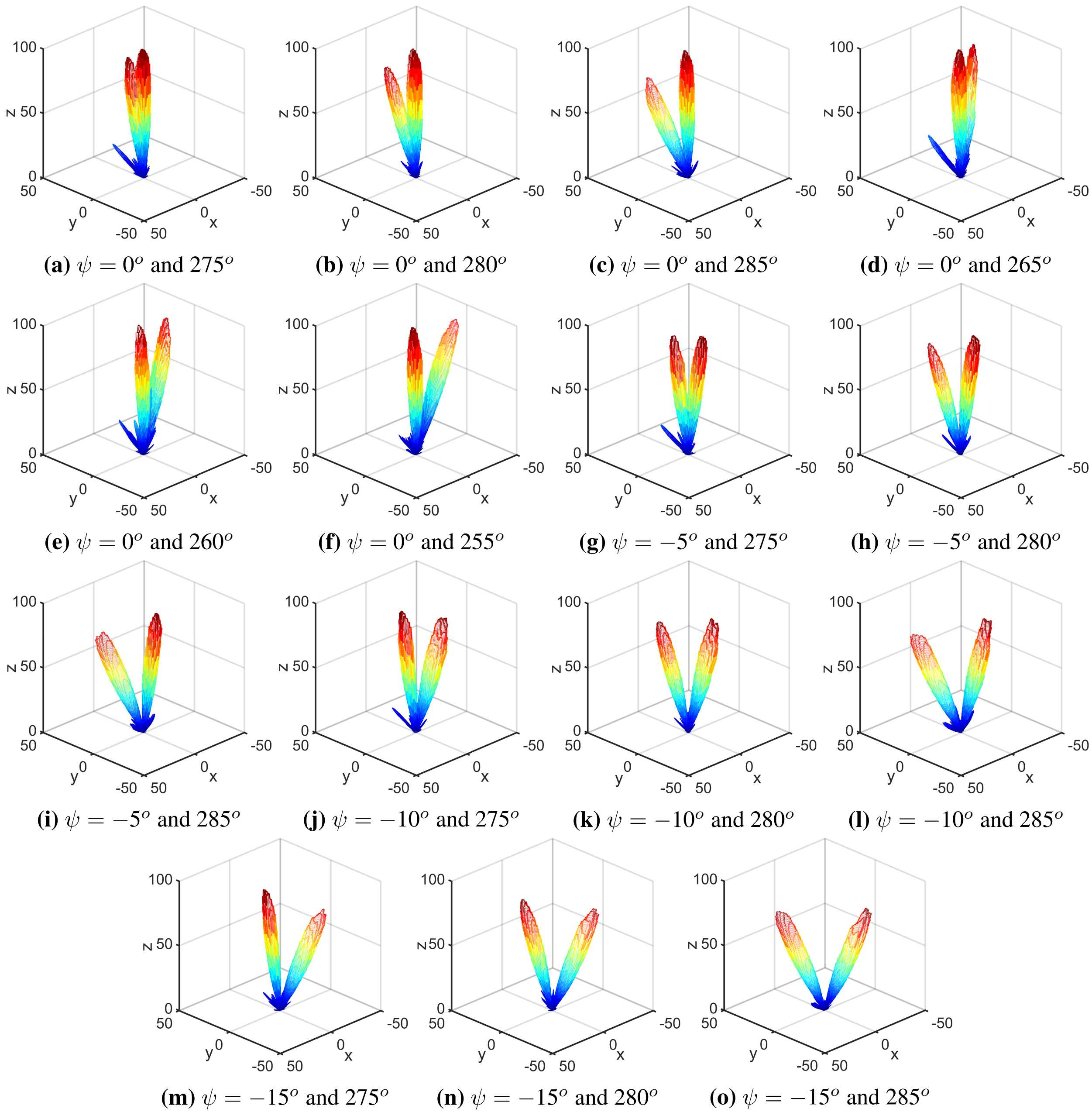

(a) $\psi = 0^o$ and 275° **(b)** $\psi = 0^o$ and 280° **(c)** $\psi = 0^o$ and 285° **(d)** $\psi = 0^o$ and 265°

(e) $\psi = 0^o$ and 260° **(f)** $\psi = 0^o$ and 255° **(g)** $\psi = -5^o$ and 275° **(h)** $\psi = -5^o$ and 280°

(i) $\psi = -5^o$ and 285° **(j)** $\psi = -10^o$ and 275° **(k)** $\psi = -10^o$ and 280° **(l)** $\psi = -10^o$ and 285°

(m) $\psi = -15^o$ and 275° **(n)** $\psi = -15^o$ and 280° **(o)** $\psi = -15^o$ and 285°

Supplementary Figure 14: Multiple-Beam 3D Plots Measurement. The normalized measured far-field 3D radiation patterns in linear scale (V/m) normalized to an arbitrary value of 100, for various values of (ψ), when both sides A and B of the 7×7 PEX array are excited simultaneously. All the beams can be independently scanned, and a multitude of radiation directions are achieved.

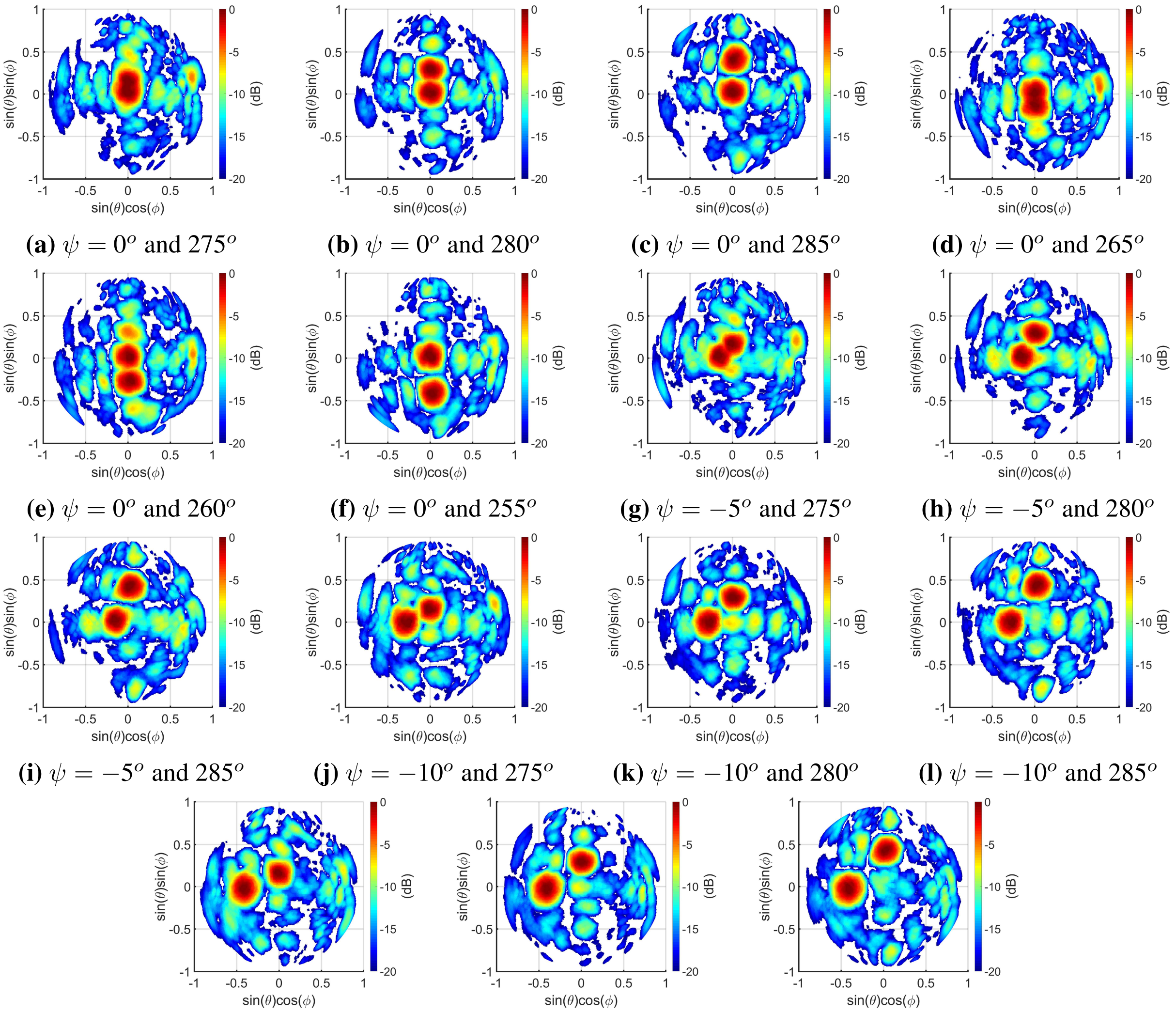

(a) $\psi = 0^o$ and 275° **(b)** $\psi = 0^o$ and 280° **(c)** $\psi = 0^o$ and 285° **(d)** $\psi = 0^o$ and 265°

(e) $\psi = 0^o$ and 260° **(f)** $\psi = 0^o$ and 255° **(g)** $\psi = -5^o$ and 275° **(h)** $\psi = -5^o$ and 280°

(i) $\psi = -5^o$ and 285° **(j)** $\psi = -10^o$ and 275° **(k)** $\psi = -10^o$ and 280° **(l)** $\psi = -10^o$ and 285°

(m) $\psi = -15^o$ and 275° **(n)** $\psi = -15^o$ and 280° **(o)** $\psi = -15^o$ and 285°

Supplementary Figure 15: Multiple-Beam U-V Plots Measurement. The normalized measured far-field U-V radiation patterns in dB scale, for various values of (ψ), when both sides A and B of the 7×7 PEX array are excited simultaneously. Both beams can be independently scanned, and a multitude of radiation directions are achieved.

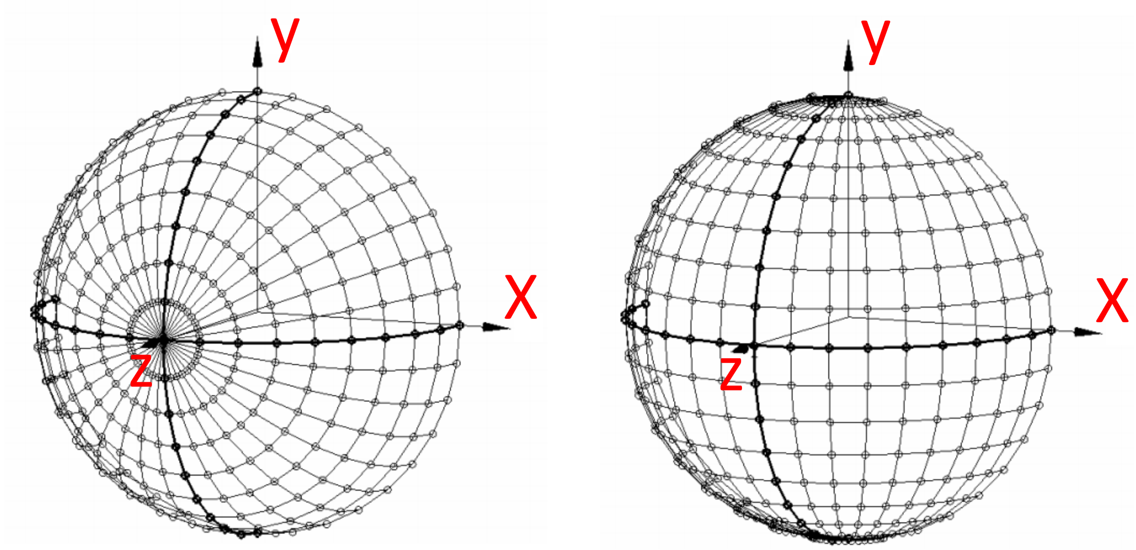

(a) Theta-Phi (θ-ϕ)

(b) Az-over-El (Az/El)

Supplementary Figure 16: Spherical Coordinate Systems. An illustration of the different planes and poles for both of the spherical coordinate systems used in this paper: **(a)** The Theta-Phi (θ-ϕ) spherical coordinate system[3]. **(b)** The Az-over-El (Az/El) spherical coordinate system[3].

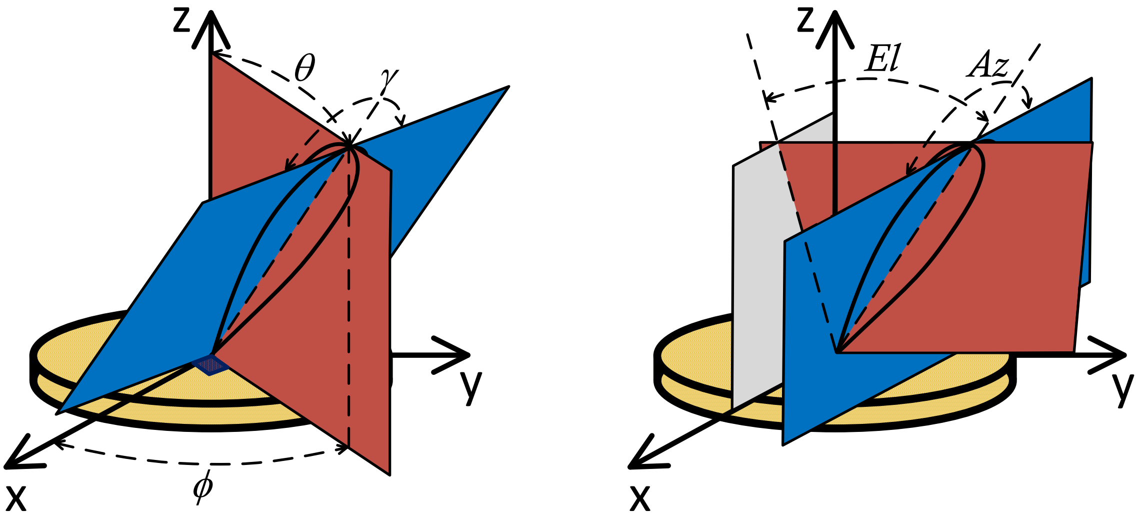

(a) Theta-Phi (θ-ϕ)

(b) Az-over-El (Az/El)

Supplementary Figure 17: Radiation Patterns Planes. (**a**) Illustration of the E-plane (blue) and H-plane (red) of a sample pencil beam generated at an azimuthal angle (ϕ) and an elevation angle (θ). For radiation patterns plotted along the E-plane, an angle (γ) is defined with zero along the peak of the generated pencil beam. (**b**) Illustration of the Az-plane (blue) and El-plane (red) of a sample pencil beam generated at an Az angle (Az) and an El angle (El).
